# Phylogenetic comparison of porcine circovirus type 2 (PCV2) and porcine reproductive respiratory syndrome virus (PRRSV) strains detected in domestic pigs until 2008 and in 2012 in Croatia

**DOI:** 10.1186/2046-0481-67-9

**Published:** 2014-05-14

**Authors:** Jelena Prpić, Tomislav Keros, Tomislav Bedeković, Dragan Brnić, Željko Cvetnić, Besi Roić, Lorena Jemeršić

**Affiliations:** 1 Croatian Veterinary Institute, Savska cesta 143, Zagreb, Croatia

**Keywords:** PCV2, PRRSV, Genetic diversity, Croatia

## Abstract

**Background:**

Porcine circovirus type 2 (PCV2) and porcine reproductive and respiratory syndrome virus (PRRSV) have been present for the last 2 decades in Croatia, causing large economical losses in the pig production. The clinical features of the infections are mostly manifested by the development of respiratory problems, weight loss and poor growth performance, as well as reproductive failure in pregnant sows. Even though the infections are continuously recognized in some regions in Croatia, the heterogeneity of the detected viral strains from 2012 has not yet been investigated. The objective of this study was to compare virus strains of PCV2 and PRRSV detected until 2008 in Croatia with strains isolated in 2012 to gain a better epidemiological understanding of these two infections.

**Results:**

PCV2 and PRRSV strains detected in 2012 in fattening pigs from regions where these two diseases have been previously described were compared to strains that have been detected in the same regions within the past two decades. The phylogenetic analysis revealed that the circulating PCV2 and PRRSV strains are distantly related to the previously described Croatian viral strains. However, when compared to known isolates from the GenBank a high genetic identity of PRRSV isolates with isolates from Hungary, Denmark and the Netherlands was found.

**Conclusion:**

The results of this study reveal that even though PCV2 and PRRSV are constantly present in the investigated regions in Croatia, the viral strains found in 2012 genetically differ from those detected in earlier years. This indicates that new entries into the pig population appeared with regard to both infections, probably as a result of pig trade.

## Background

Porcine circovirus (PCV) is a small, non-enveloped virus with a single-stranded circular DNA of 1.76 kb [1,2] belonging to the genus *Circovirus* within the family *Circoviridae*, along with other animal viruses such as chicken anaemia virus and beak and feather disease virus [3]. PCV genome contains three open reading frames (ORF), ORF1 encoding a replicase, ORF2 encoding the capsid protein and ORF3 encoding a protein related to cell apoptosis [4]. Two types of PCV have been described, namely PCV1 and PCV2. Their DNA sequence homology is 68 to 76% [5]. While PCV1 is not pathogenic, PCV2 has been recognised as the causative agent of several pathological syndromes in swine causing great economical losses in the pig industry worldwide. PCV2 isolates are genetically divided into three groups, PCV2a, PCV2b and PCV2c, isolates of most of which are distributed among groups PCV2b and PCV2a, respectively [6,7]. Boisseson et al. [8] found that the variations among the PCV2 genomic sequences are mainly due to the variability within ORF2 (91.2-100% nucleotide identity), while the ORF1 is highly conserved (97.8-100% nucleotide identity).

Porcine reproductive and respiratory syndrome virus (PRRSV) is a small enveloped positive-stranded RNA virus that belongs to the *Arteriviridae* family, genus *Arterivirus*[9]. Its genome is composed of approximately 15 kb [9] containing 9 ORF’s [10,11]. According to the genome characteristics, PRRSV is divided into two genotypes, the European, or type 1 virus, also reffered to as the Lelystad virus (LV), and the American, or type 2 virus. They share a sequence identity of 50-70% [12,13]. Since PRRSV has the highest calculated rate of nucleotide substitution reported so far for a RNA virus, a significant genetic heterogeneity exists even within the two main groupings and the sequence analysis allows sub-typing of PRRSV to geographical levels [12,14]. According to a new proposal, based on recent sequence information, the European PRRSV-1 genotype should consist of 4 subtypes [14].

The wide variability of PRRSV strains causes several potential problems in the diagnosis of PRRS [15]. Therefore, molecular methods accompanied by sequencing represent a more sensitive and specific tool in PRRS diagnosis. Genetic analysis of ORF7 is mostly used for revealing the genetic relationships among PRRSV strains [16].

Both, PRRS [17] and PCV2 [18] infections have been described in Croatia in 1997 and 2004, respectively, on large, small and medium size pig farms in several Croatian counties. Since then, limited data is available on the predominant genotypes and eventual genetic changes of both viruses, especially after introducing vaccination.

Therefore, the objective of this study was to compare isolates of PCV2 and PRRSV collected until 2008 with the newly detected strains from 2012 that have been the cause of severe losses in the pig industry in Croatia. Direct sequencing of the amplified part of PCV2 ORF1 and PRRSV ORF7 was performed to determine genetic heterogeneity of the virus isolates and to carry out the comparison with previously described isolates.

## Materials and methods

### Sample collection

Serum samples from domestic pigs 3 to 6 months of age with severe signs of PCV2 clinical disease from two large pig farms located in Osijek-Baranja County with a history of PCV2 infection were collected during 2012. Prior to the extraction of total DNA, samples were pooled (5–10 samples per pool) and kept frozen at −70°C. A total of 10 pools, 5 cointaining samples collected from one PCV2 affected farm (CRO-PCV2-1 to CRO-PCV2-5) and 5 pools from the second PCV2 affected farm (CRO-PCV2-6 to CRO-PCV2-10) were examined for the presence of PCV2 DNA.

In addition, serum samples collected from domestic pigs with clinical signs of PRRS from large farms located in Brod-Posavina as well as Osijek-Baranja County were also included in this study.

Prior to the extraction of total RNA, samples that originated from 3–6 month old pigs from the same farm were pooled and kept frozen at −70°C. A total of 11 pools containing 5–10 samples each from Brod-Posavina (samples CRO-PRRSV-1 to CRO-PRRSV-3) and Osijek-Baranja County (samples CRO-PRRSV-4 to CRO-PRRSV-11) were examined for the presence of both viruses, PCV2 and PRRSV.

### Sample preparation and viral DNA and RNA extraction

Sera were separated from cellular elements by centrifuging coagulated blood (the blood clots were rimmed with a sterile glass stick to facilitate separation) for 15 min at 1000 × g. An amount of 140 μl of each serum sample was used for viral RNA purification by using the QIAamp® Viral RNA Mini Kit (Qiagen, USA) according to the manufacturer’s instructions. An amount of 200 μl of each serum sample was used for viral DNA purification by using the QIAamp® DNA Mini kit (Qiagen, USA) according to the manufacturer’s instructions.

### Detection of PCV2 by polymerase chain reaction

For detection of PCV2 DNA a specific primer pair for the amplification of a 360 bp long sequence of ORF1 region [19] was used. The reaction mixtures (50 μl) consisted of 6 μl of viral template DNA, 5 μl of 10x PCR buffer, 1.5 mM MgCl_2,_ 200 μM of each deoxinucleotide triphosphates (dNTP), 400 nM of each primer, 5U of Platinum Taq DNA polymerase (Invitrogen, USA) and nuclease free water. Reaction mixtures without template DNA were used as negative controls. Croatian PCV2-positive samples were used as positive controls. The thermal profile of the amplifications contained an initial denaturation step at 94°C for 2 min, which was followed by 30 cycles of denaturation at 94°C for 30 sec, annealing at 56°C for 30 sec, and primer extension at 72°C for 30 sec. The amplifications were finished by a final extension at 72°C for 3 min. Eventually, the reaction mixtures were maintained at 4°C until they were removed from the device.

### Detection of PRRSV by RT-PCR

For detection of PRRSV RNA a specific primer pair positioned in the ORF7 region (660 bp) [20] was used. RNA samples were reverse transcribed by SuperScript III reverse transcriptase and random hexamers (Invitrogen Life Technologies, USA), following the manufacturer’s instructions. The generated cDNA was used immediately for PCR amplification or stored at −20°C. The reaction mixtures were prepared as described for PCV2 mixtures. Reaction mixtures without template RNA were used as negative controls. Croatian PRRSV-positive samples were used as positive controls.

The thermal profile of the amplifications contained an initial denaturation step at 94°C for 3 min, which was followed by 30 cycles of denaturation at 94°C for 45 sec, annealing at 55°C for 45 sec, and primer extension at 72°C for 45 sec. The amplifications were finished by a final extension at 72°C for 7 min. Eventually, the reaction mixtures were maintained at 4°C until they were removed from the device.

### Gel electrophoresis

Amplification products were separated by agarose gel electrophoresis in 1.5% agarose gel stained with ethidium bromide and visualized by UV transillumination.

### Nucleotide sequencing and phylogenetic analysis

Prior to sequencing PCR products were excised from 2% agarose gels and then purified using Wizard SV Gel and PCR Clean-Up System (Promega, USA). Purified samples were sent for direct sequencing in both directions to Macrogen Inc; Amsterdam, the Netherlands.

The first comparisons of sequence data with the reference strains from the GenBank were performed by algorithm BLAST (http://blast.ncbi.nlm.nih.gov/Blast.cgi). For nucleotide sequence determination chromatograms were analysed by the Sequencer 4.6. programme (http://www.genecodes.com, Genes Codes Corporation). Sequences characterized in this study were aligned with published reference strains using the ClustalX programme. For the reconstruction of phylogenetic trees the Neighbor-Joining (NJ) method with Kimura-2 Parameter Model followed by MEGA 5 were used. The clustering stability of the NJ tree was evaluated by bootstrap analysis with 1000 replicates. The GenBank accession numbers of all reference sequences used for phylogenetic comparisons are indicated in Table 1. All PCV2 and PRRSV sequences displaying nucleotide differences obtained in the present study were deposited in the GenBank under accession numbers KF498717-KF498720 (CRO_PCV2_1, 3, 4, 6) and KF498721-KF498727 (CRO_PRRSV_1, 2, 3, 4, 7, 8, 9).

**Table 1:** PCV2 and PRRSV reference sequences used for phylogenetic analysis retrieved from the GenBank

**GenBank accession no.**	**Original name**	**Country**
HQ591366	PCV2 , isolate 90-08-21	Croatia, 2008
HQ591367	PCV2, isolate 110-08-2	Croatia, 2008
AY256460	PCV2, strain 375	Hungary, 2003
AY424405	PCV2, isolate AUT5	Austria, 2003
HQ591368	PCV2, isolate 126-07-5	Croatia, 2007
HQ591369	PCV2, isolate 147-07-7	Croatia, 2007
HQ591370	PCV2, isolate 161-08-2	Croatia, 2008
HQ591365	PCV2, isolate 70-08-2	Croatia, 2008
AY180397	PCV2, strain Pingtung-5	Taiwan, 2002
AY484410	PCV2, isolate NL-control-4	Netherlands, 2003
AF311296	BFDV	Australia, 2000
AF071879	PCV1	SAD, 1998
GU930366	PRRSV, isolate Hun06	Hungary, 2009
AY035960	PRRSV, isolate 361-4	Denmark, 1994
JQ043210	PRRSV, 174-07-1	Croatia, 2007
DQ324711	PRRSV, isolate Prz-87	Poland, 2005
JQ043212	PRRSV, isolate 660-07-2	Croatia, 2007
JQ043213	PRRSV, isolate 1152-08-10	Croatia, 2008
AY035976	PRRSV, strain 2567/96	Italy, 1996
AY035961	PRRSV, isolate 48/92-1	Denmark, 2001
AY035975	PRRSV, isolate 2481/97	Denmark, 2001
M96262	Lelystad virus	Netherlands, 1991
JQ043211	PRRSV, 177-07-2-1	Croatia, 2007
GU930368	PRRSV, isolate Hun08	Hungary, 2009
DQ324708	PRRSV, strain Okt-46	Belarus, 2004
DQ324719	PRRSV, strain Soz-6	Belarus, 2004
DQ324727	PRRSV, strain Yuz-34	Belarus, 2004
EU071271	PRRSV, strain KR	Russia, 2007
AF438362	PRRSV, strain Aus	Lithuania, 2000
DQ324701	PRRSV, strain Bor-41	Belarus, 2004
DQ324702	PRRSV, strain Bor-54	Belarus, 2004
L39363	PRRSV, isolate IL1	United States, 1996
L39362	PRRSV, isolate IA6	United States, 1996
U18750	PRRSV, isolate ISU-3927	United States, 1996

## Results

The amplification of PCV2 genome regions from samples originating from the two farms with clinical signs of PCV2 (CRO-PCV2-1 to CRO-PCV2-10) resulted in clear PCR products with the expected molecular size. RT-PCR assay, using a primer pair specific for a sequence within the PRSSV ORF7, resulted in strong amplification signals with the expected molecular size in all pooled samples collected from swine with clinical signs of PRRS (CRO-PRRSV-1 to CRO-PRRSV-11).

No co-infections with these two viruses were detected in the tested samples. Amplifications were never detected in negative controls.

**Figure 1:**
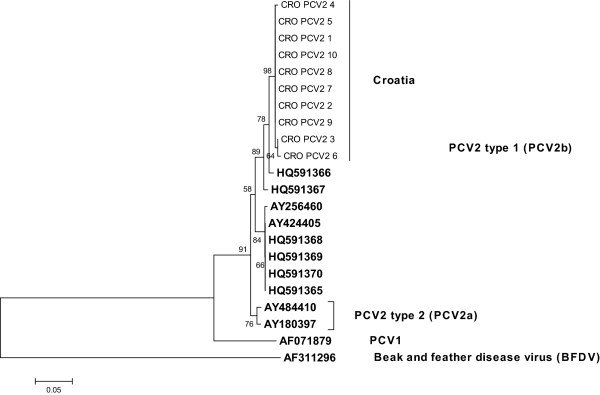
**Neighbor-joining phylogenetic tree obtained by the analysis of the partial ORF1 region (297 bp) of Croatian PCV2 samples.** Reference sequences included in the analysis are marked in bold. Bootstrap values are presented next to tree nodes. The bar represents 0.05 nucleotide substitution per site.

The PCR products of 360 bp were sequenced and confirmed to be PCV2 specific. The phylogenetic relationship analysis of the obtained sequences, including referent ones, showed that all analysed PCV2 sequences clustered into phylogenetic group 1 or PCV2b group (Figure 1). The seven PCV2 sequences (CRO-PCV2-1, CRO-PCV-2, CRO-PCV2-5, CRO-PCV2-7 to CRO-PCV2-10) were found to be 100% identical among themselves in the 297 nt ORF1 region. Sequences CRO-PCV2-3 and CRO-PCV2-4 differed in one (0.33%), whereas sequence CRO-PCV2-6 differed in two (0.66%) nucleotides. The robustness of the tree topology was supported by high bootstrap values. The obtained phylogenetic clustering shows that the PCV2 isolates differ from the published Croatian isolates from the year 2008 (HQ591366, HQ591367) in 2.3% and 3.0% of nucleotides, respectively. They differ in 4.4% nucleotides from the published Croatian isolates HQ591365, HQ591370 (from the year 2008), HQ591368 and HQ591369 (from the year 2007).

**Figure 2:**
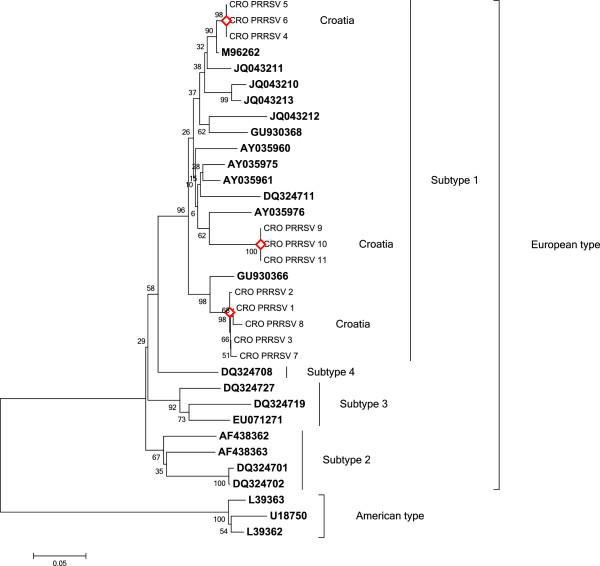
**Neighbor-joining phylogenetic tree obtained by the analysis of the partial ORF7 region (375 bp) of Croatian PRRSV samples.** Reference sequences included in the analysis are marked in bold. Bootstrap values are presented next to tree nodes. The bar represents 0.05 nucleotide substitution per site.

The PCR products of 660 bp were sequenced and confirmed to be PRRSV specific. The phylogenetic relationship analysis of the obtained sequences, including referent ones, showed that all analysed PRRSV sequences clustered into European type (type 1), subtype 1 (Figure 2). Sequences CRO-PRRSV-4, CRO-PRRSV-5 and CRO-PRRSV-6 (group 1) were found to be 100% identical among themselves in the 375 nt ORF7 region, while sequences CRO-PRRSV-9, CRO-PRRSV-10 and CRO-PRRSV-11 (group 2) were 100% identical. Sequences CRO-PRRSV-1, CRO-PRRSV-2, CRO-PRRSV-3, CRO-PRRSV-7 and CRO-PRRSV-8 (group 3) differed in one (0.26%) to six (1.60%) nucleotides. Sequences belonging to group 1 differed from the sequences belonging to group 3 in 31 (8.26%) to 35 (9.33%). The lowest sequence identity was found in sequences from group 2 when compared to the ones from group 3 where 39 (10.40%) to 43 (11.47%) nucleotides differed. Sequences from group 1 differed from the sequences from group 2 in 37 (9.87%) nucleotides. The obtained phylogenetic clustering shows that the PRRSV isolates differ from the published Croatian isolates from the year 2007, JQ043211 in the range of 3.7-9.2%, JQ043210 in the range of 4.7-10.0%, JQ043212 in the range 8.0-11.7%. They differ from the Croatian isolate from the year 2008 (JQ043213) in the range of 4.7-9.0%. The Croatian PRRSV isolates from 2012 showed a high genetic identity with Danish (92.78%), Hungarian (96.51%) and Dutch (99.00%) isolates.

## Discussion and conclusions

Due to their potentially high economic impact, PRRSV [21] and PMWS [22] are two of the most important diseases affecting pig production in swine producing countries. It is thought that the PRRSV and PCV2 (the causative agent of PMWS) have been present in pig herds for decades if not centuries before the diseases emerged, and the viruses slowly mutated into disease-causing forms. Evidence from field surveys suggests that co-infections of pigs with these two viruses is common [23]. Preliminary studies have shown that both PCV2 and PRRSV were present in the livers of sows with PRRS-associated hepatitis and both PRRSV and PCV2 antigen or genome have been detected in tissues from the majority of PMWS cases [24]. However, the detection of PCV2 and PRRSV in the same animals may only be co-incidental since both are present in a high prevalence in commercial pig populations. In our study, no co-infection has been found.

Porcine circovirus type 2 (PCV2) has been detected in pigs with various clinical conditions on pig farms in different regions in Croatia [18]. The signs that predominated in the affected pigs were skin lesions, loss of weight, respiratory and digestive disorders followed by an increase in the mortality rate. Phylogenetic analyses from the year 1997 and 2002 showed clustering of PCV2 isolates into PCV2a and PCV2b groups, respectively [18]. Isolates from the year 2012 clustered into the PCV2b group and were related to those from year 2002, even though nucleotide differences occur suggesting the appearance of novel strains. No isolates from group PCV2a were found in 2012 indicating that this viral group has spontaneously decreased in its spread during the last decade or has been eliminated from the tested farms as a result of vaccination. Similar findings were reported and recognised in countries neighbouring Croatia, such as Slovenia [25], Hungary [26], Austria [27], Serbia [28,29], and Italy [30]. Vaccination against PCV2 infection in Croatia was introduced rather late, in 2010. Only commercially available inactivated and subunit vaccines are registered, therefore no chimeric PCV2 were expected to be found during this study, as it was recorded in Canada [31].

First cases of reproductive failures of higher intensity caused by PRRSV on several pig farms in Croatia date from the year 1995 [17]. The same clinical signs of acute respiratory disease, mortality among growing pigs, reproductive and/or respiratory disorders in sows were reported at the same time in countries neighbouring Croatia, such as Slovenia [15], Serbia [32], Hungary [26,33], and Austria [34]. Phylogenetic analysis of strains detected in the year 2007 showed clustering of PRRSV isolates into the European type [35]. The same results have been gained when isolates from Croatia collected in 2012 were genetically analysed. However, new subgroups of PRRSV strains were identified in 2012 when compared to the ones previously described in Croatia. Therefore, a possibility of new PRRSV entries within the last decades is possible. The high genetic identity shown among the 2012 PRRSV isolates from Croatia and isolates from Hungary, Denmark and the Netherlands could indicate that the novel strains have entered through trade. Vaccination against PRRS started in Croatia in 2004. It was massively applied until 2008. Two commercially available vaccines are registered, of which only one (based on an attenuated PRRSV strain) was actually in use. However, vaccination has drastically decreased after 2008 and only 750 doses were sold in 2012 and 1500 in 2013. All isolates in this study were sequenced and vaccinal strains were not found.

The detection of new PCV2 and PRRSV isolates in this study expanded the known diversity of detected strains in Croatia. This study also confirmed that the molecular-based reverse transcription polymerase chain reaction method and the routine direct sequencing of PCR products are important tools for rapid recognition of new PRRSV field strains.

## Competing interests

The authors declare that they have no competing interests.

## Authors’ contributions

JP: Designer and performer of the study and author of the manuscript; TK, TB, DB, ŽC, BR, LJ: Contributed to the design, performing and revising of the manuscript. All authors read and approved the final manuscript.
